# Theory of planned behaviour variables and objective walking behaviour do not show seasonal variation in a randomised controlled trial

**DOI:** 10.1186/1471-2458-14-120

**Published:** 2014-02-05

**Authors:** Stefanie L Williams, David P French

**Affiliations:** 1 Applied Research Centre in Health and Lifestyle Interventions, Faculty of Health and Life Sciences, Coventry University, Priory Street, Coventry CV1 5FB, UK; 2 Manchester Centre for Health Psychology, School of Psychological Sciences, University of Manchester, Coupland 1 Building, Oxford Road, Manchester M13 9PL, UK

**Keywords:** Season, Walking, Randomised controlled trial, Theory of planned behaviour, Behaviour change

## Abstract

**Background:**

Longitudinal studies have shown that objectively measured walking behaviour is subject to seasonal variation, with people walking more in summer compared to winter. Seasonality therefore may have the potential to bias the results of randomised controlled trials if there are not adequate statistical or design controls. Despite this there are no studies that assess the impact of seasonality on walking behaviour in a randomised controlled trial, to quantify the extent of such bias. Further there have been no studies assessing how season impacts on the psychological predictors of walking behaviour to date. The aim of the present study was to assess seasonal differences in a) objective walking behaviour and b) Theory of Planned Behaviour (TPB) variables during a randomised controlled trial of an intervention to promote walking.

**Methods:**

315 patients were recruited to a two-arm cluster randomised controlled trial of an intervention to promote walking in primary care. A series of repeated measures ANCOVAs were conducted to examine the effect of season on pedometer measures of walking behaviour and TPB measures, assessed immediately post-intervention and six months later. Hierarchical regression analyses were conducted to assess whether season moderated the prediction of intention and behaviour by TPB measures.

**Results:**

There were no significant differences in time spent walking in spring/summer compared to autumn/winter. There was no significant seasonal variation in most TPB variables, although the belief that there will be good weather was significantly higher in spring/summer (F = 19.46, p < .001). Season did not significantly predict intention or objective walking behaviour, or moderate the effects of TPB variables on intention or behaviour.

**Conclusion:**

Seasonality does not influence objectively measured walking behaviour or psychological variables during a randomised controlled trial. Consequently physical activity behaviour outcomes in trials will not be biased by the season in which they are measured. Previous studies may have overestimated the extent of seasonality effects by selecting the most extreme summer and winter months to assess PA. In addition, participants recruited to behaviour change interventions might have higher levels of motivation to change and are less affected by seasonal barriers.

**Trial registration:**

Current Controlled Trials ISRCTN95932902

## Background

The Department of Health recommends that individuals engage in at least 150 minutes of moderate intensity, or 75 minutes of vigorous intensity, physical activity (PA) across the week to gain protective health benefits [1]. Only 39% of men and 29% of women in England meet these recommendations [2]. Brisk walking (i.e. walking at a sufficient intensity to result in the walker getting out of breath) is a promising target for physical activity interventions due to its acceptability and accessibility [3]. There are also substantial health benefits associated with walking [4-7]. Therefore developing effective interventions to promote walking behaviour is of considerable public health benefit. Furthermore, it is essential that steps are taken to minimise bias in randomised controlled trials of interventions to promote walking.

The effectiveness of interventions to promote physical activity, and specifically walking behaviour, might be influenced by the season in which they are delivered, with physical activity levels at their highest in the summer and lowest in winter. The impact of seasonality on PA behaviour in general has been reported extensively in the literature [8-10]. A systematic review of studies from eight different countries, including over 291,000 participants, supported the hypothesis that levels of physical activity vary according to season with physical activity levels being highest in the summer months [11]. None of the studies included in the systematic review, or in more recent studies [9,10], reported on the effect of seasonality as part of an intervention or trial. Instead all studies used deliberate sampling to assess seasonal variability in physical activity, with participants typically completing surveys or wearing pedometers at specific seasonal time points i.e. once in summer and once in winter.

Previous studies of seasonality and walking have reported similar fluctuations in walking behaviour between summer and winter seasons [12-14]. Hamilton et al. [14] investigated whether pedometer step counts varied between summer and winter, with the aim of rectifying sampling and measurement problems apparent in previous studies [12,13]. Ninety-six UK adults wore a pedometer at two measurement periods; summer data were collected between June and September and winter data were collected between January and March. A seasonal difference in step counts was observed, with summer step counts significantly higher than winter step counts. Furthermore, Clemes and colleagues [15] also reported significant reductions in step counts from summer to winter. This reduction was more apparent for normal weight individuals than overweight individuals. A recent longitudinal observational study investigated the impact of seasonality on accelerometer measures of physical activity at different intensities [16] with data collected across all four seasons; spring, summer, autumn and winter. This study found that seasonality impacted on light-intensity physical activity only as measured by an accelerometer, with light intensity physical activity higher in spring and summer. However, no significant effect of season on the mean proportion of time spent engaging in moderate-vigorous activity was found.

Whilst the authors of the above studies discuss the implications of their findings in terms of the possibility of seasonally tailoring physical activity interventions, an important omission in the literature is that the impact of such seasonal variation in randomised controlled trials has not been studied. Given that one purpose of randomised controlled trials is to limit the influence of such confounding variables, it is surprising that season has been neglected as a potential confounder of such studies. Furthermore, in a recent systematic review of trials of walking interventions seasonality was not reported as a variable that might influence effectiveness [17]. There is therefore a gap in the literature regarding the influence of seasonality in randomised controlled trials.

Given that trials are aiming to change behaviour, the behaviour of participants is likely to be different in comparison to simple longitudinal studies with no intervention component. Thus it is possible that the effects of season might be dampened in a trial situation compared to the studies detailed earlier [14,15]. Alternatively, if seasonality is to have an effect on objective walking behaviour in a randomised controlled trial this has the possibility to bias trial results, particularly in studies with a follow up of less than 12 months duration. Of the 48 studies included in the Ogilvie review [17], 32 studies included a follow up of less than 12 months duration. This will therefore have important implications in terms of the accuracy of the current literature regarding interventions to promote walking behaviour. Furthermore, a previous study has evaluated the impact of seasonality on recruitment of participants to clinical trials [18] and found peaks in recruitment in spring. However, its impact in terms of the outcome under investigation has not yet been clarified.

Only one study to our knowledge has assessed the relationship between weather variation and walking behaviour as part of an intervention study [19], although it had no control group, and had only twenty-five participants. In this study sedentary adults were recruited to the First Step Program (FSP), a behaviour change intervention based on Social Cognitive Theory. Weather had a modest impact on step counts in this study, with rainfall and snow negatively affecting step counts. Increases in temperature were associated with a positive change in step counts corresponding to an average 2.9% increase for every 10 degree increase in temperature. Over the differing weather variables reported there was a 1%-20% drop in step counts from summer to winter. However, this study was conducted in Canada, therefore its findings cannot be generalised to other countries with more temperate climates such as the UK due to substantial weather variation between the two countries. For example, average winter temperature in the Canadian study [19] was -9.9˚C (January) whilst average UK winter temperatures vary between 0.9˚C and 6.4˚C in the same month [20].

Another gap in the literature is that seasonal variation on the determinants of walking behaviour has not been considered. Where there are seasonal changes associated with walking behaviour, it is likely that the same relationship exists between season and the theoretical determinants of the behaviour. It is of importance that the theoretical determinants of walking behaviour, and the social and environmental influences on such determinants, are investigated in order to develop optimal behaviour change interventions [21].

The Theory of Planned Behaviour [22] is a good candidate theory for examining theoretical determinants of behaviour. It has been used to predict physical activity in over one hundred studies [23], as well as other behaviours in over a thousand studies [24]. The TPB has also consistently been demonstrated to be an important predictor of physical activity intentions and behaviour [23-25]. The TPB has also been shown to be a significant predictor of intentions to walk more in a recent review [26] as well as walking behaviour measured by self-report [27] and objective measures [28]. Despite this wealth of research, there are no published studies to our knowledge that have assessed whether seasonality has a similar impact on Theory of Planned Behaviour [22] variables to that of physical activity behaviour.

According to the TPB the proximal determinants of behaviour are a person’s intention to perform that behaviour and their perceived behavioural control (PBC) i.e. a person’s belief that performance of the behaviour is within his/her control [29]. Intention itself is proposed to be determined by three variables; attitude, subjective norms, i.e. perceptions of social pressure and PBC [22].

It can be hypothesised that seasonal variation would impact on an individual’s PBC in particular, as weather-related changes associated with different seasons could be considered barriers to perform the behaviour. As such this will impact on the perceived ease and difficulty with which an individual can perform the behaviour. According to the theory, PBC is in turn determined by control beliefs, which refer to an individual’s belief regarding the presence of factors that facilitate or inhibit behaviour. In combination with its associated perceived power to inhibit/facilitate behaviour, a control belief has an indirect effect on an individual’s PBC. It can also be hypothesised that control beliefs regarding weather in particular will impact on an individual’s perceived behavioural control regarding walking, particularly in the winter season.

The present study therefore aims to assess seasonal differences in a) objective walking behaviour and b) TPB variables during a randomised controlled trial of an intervention to promote walking.

## Methods

### Design

The data reported in the present study are drawn from a two-arm cluster randomised controlled trial of an intervention to promote walking in primary care [30]. For the present study, a within-subjects design was employed, which consisted of a series of repeated comparisons of “spring/summer” and “autumn/winter” measures of objective walking behaviour and Theory of Planned Behaviour variables, collected immediately post-intervention and at 6-months follow up. Completing the planned analyses using four seasons for comparison was not appropriate for the current study due to the limited cases in some groups (i.e. time 4 winter), thus the study would have been insufficiently powered to detect significant findings. Therefore data from the four seasons were collapsed into the two groups stated above.

Solstice and Equinox dates for the period of data collection, between 3rd August 2010 and 15th December 2011, were used to determine spring/summer and autumn/winter dates. Data collected between 20th March and 22nd September was coded as “spring/summer”, and data collected between September 23rd and March 19th was coded as “autumn/winter”. This criterion for defining seasons has been used in previous studies investigating the effect of seasonality on walking behaviour [14,15].

### Participants

Participants were recruited in a sequential process from GP practices in a geographically and socially-diverse sub region of central England. Twenty-one GP practices were recruited to the study. Further information on the recruitment process can be found elsewhere [30].

Participants were eligible for inclusion in the study if they were a) aged between 16 and 65, b) had one or more chronic conditions for which increasing physical activity would have a positive effect on health status (e.g. hypertension, cardiovascular disease, diabetes, obesity), and c) were inactive in terms of not meeting government physical activity guidelines.

### Procedure

Participants received one of two interventions, a) a self-regulation walking intervention or b) an information provision plus pedometer.

Patients completed a Theory of Planned Behaviour questionnaire immediately before (t1) and after receiving one of the two interventions (t2), at six weeks following receipt of the intervention (t3), and at six months post-intervention (t4). TPB questionnaires were completed immediately prior to the participant wearing a pedometer at each time point.

All participants were asked to wear a pedometer for seven days immediately after (t2) receiving one of the two interventions, and to return it at the end of this period in a reply-paid envelope. Participants were sent another pedometer 6 weeks (t3) and 6 months (t4) after the intervention, and asked to wear this for seven days and return in a reply-paid envelope at each of these time points.

### Measures

Participants wore a New Lifestyles NL-1000 pedometer (New-Lifestyles Inc, Lees Summit, Missouri, USA) at each time point to assess objective walking behaviour. The NL-1000 pedometer has been shown to be a highly accurate research grade pedometer in recent validity assessments, based on both free-living and laboratory experiments [31]. The intensity level was set at level 4–9 in the current study in order to record moderate and vigorous activity only, walking activity at an intensity level below this was not recorded. This pedometer is suitable for use with participants at all BMI levels. Time spent walking per day and step count data was recorded using the NL-1000 pedometer.

A six-item TPB questionnaire was completed by all participants immediately post-intervention (t2) and at 6-weeks post-intervention (t3). The six-item questionnaire included one attitude item (“Walking for 30 minutes on average a day over the next 7 days will be Unpleasant/Pleasant”), one subjective norm item (“Most people who are important to me will themselves walk for 30 minutes on average a day over the next 7 days”) and one intention item (“I intend to walk for 30 minutes on average a day over the next 7 days”). Three PBC items were included (“If I wanted to I could walk for 30 minutes on average a day over the next 7 days”, “How much control do you believe you have over walking for 30 minutes on average a day over the next 7 days”, “It is mostly up to me whether or not I walk for 30 minutes on average a day over the next 7 days”). This shortened questionnaire was based on one previously developed [32] and validated [28] in studies of walking behaviour with adult volunteer samples.

A 26-item version of this TPB questionnaire was completed by all participants in the present study prior to receiving the intervention (t1) and at 6-months post-intervention (t4). This questionnaire included three attitude, subjective norm, PBC and intention questions (All Cronbach’s α = .78-.86). The full 26-item TPB questionnaire included five items to measure control beliefs in relation to walking for at least 30 minutes on average a day over the next 7 days [33] and associated perceived power to inhibit/ facilitate behaviour in relation to walking for at least 30 minutes on average a day over the next 7 days. The single-item and three-item measures of attitude (r = .84), subjective norm (r = .81) and intention (r = .87), delivered at baseline, were all highly correlated (all p < .001). Personal data on age, gender and BMI was recorded by the practice nurse or healthcare assistant at the patients’ initial appointment. Ethnicity, employment status, education level was stated by the patient using standardised questionnaire measures at the same appointment.

### Meteorological information

Regional summary weather data was retrieved retrospectively from the Met Office following data collection [20]. Data summarising the mean, minimum and maximum temperature (°C), volume of monthly rainfall (mm), and number of hours of sunshine were retrieved from the Met Office website for the period of data collection described above.

### Statistical analysis

Time spent walking was measured by a New Lifestyles NL-1000 pedometer (New-Lifestyles Inc, Lees Summit, Missouri, USA), set at an intensity level 4–9 (moderate and vigorous activity). Walking activity at an intensity level below this was not recorded. Missing time spent walking data were imputed based on the baseline characteristics of the practice and individual, using predictive mean matching [34]. Where less than five minutes walking was recorded for any particular day, the reading for that day was treated as missing, and not used in calculating mean daily duration of walking. When individual days’ data were missing, the mean was taken over these non-missing days as long as data were available for two or more days. If no data, or data from fewer than two days, were available for a particular patient at a particular time point, that assessment of walking was treated as missing.

A series of repeated measures analyses of covariance (ANCOVAs), controlling for intervention group, were conducted to assess the main effect of season on objective walking behaviour and TPB variables (attitudes, subjective norms, PBC, intention). Measures taken at t2 and t4 were used, as these two time-points were six months apart, allowing for a comparison of spring/summer and autumn/winter months.

Repeated measures ANCOVAs were applied to each control belief, and associated power, separately i.e. time, work/family commitments, pain, weather, threatening areas. Control beliefs were measured at only two time points, baseline (t1) and immediately-post intervention (t4), therefore seasonal analysis was conducted using data from these time points.

A sensitivity analysis was used to assess the robustness of the main findings of the present study. To assess this a complete case analysis of step count data retrieved in the present study was conducted using a repeated measures ANCOVA. Step counts were recorded using the NL-1000 pedometer, where the pedometer recorded less than 1000 steps for a particular day this case was treated as missing.

To assess whether season moderated the prediction of intention and objective walking behaviour, a series of multiple hierarchical regression analyses were conducted. For the regression analyses, intervention group was included at step one as is good practice for running cohort analysis on trial data [35] and demographics were included at step two. Season was included in the model as the next step.

Where intention was the dependent variable, attitude, subjective norm and PBC were next entered into the regression model. An interaction term was created between season and each TPB variable separately, and each interaction term was entered into the regression model in the final step. To create the interaction term each independent variable was converted to a deviation score, resulting in a mean of zero, in order to reduce problems with multicollinearity [36,37].

Where behaviour was the dependent variable, intention and PBC were instead entered into the model at level four. An interaction term between season and intention, and PBC, was created using the same method as described above and included in the final step of this regression model. Statistical analyses were conducted using SPSS for windows version 20.0 (SPSS Inc, Chicago, IL, USA).

## Results

### Participant characteristics

Three hundred and fifteen patients received one of the two interventions, n = 136 received the self-regulation intervention and n = 179 received information provision. The intervention groups did not significantly differ on objective walking behaviour at any time point during the trial. There were significant differences in intention, PBC and subjective norm between intervention groups immediately post-intervention (all p < .05), and significant differences in subjective norm between groups at 6-weeks and 6-months (p = .013, and .016, respectively). Intervention group was therefore controlled for in all analyses.

The mean age of participants in the trial was 55.2 (9.30) years, 64.8% were female and 35.2% were male, and the average BMI was 30.3 kg/m^2^ (SD = 5.4). Eighty four percent of participants were British White, 29% were employed full-time, and 25% held a degree or postgraduate qualification.

### Seasons

Immediately post-intervention (t2) 69.8% (n = 220) of questionnaires were completed in autumn/winter, and 24.4% were completed in spring/summer (n = 77). Pedometers were returned by n = 204 participants in autumn/winter, and n = 69 in spring/summer at this time point.

At six-month post-intervention (t4) n = 58 (18.4%) of questionnaires were completed in autumn/winter, and n = 176 (55.9%) were completed in spring/summer. Pedometers were returned by n = 56 (17.8%) participants in autumn/winter, and n = 174 (55.2%) were returned in spring/summer.

### Meteorological information

Meteorological data for the 'spring/summer’ and 'autumn/winter’ monitoring periods are summarised in Table 1. Mean, and minimum and maximum, temperatures (˚C) and sunshine hours were markedly lower in 'autumn/winter’ than in 'spring/summer’ , as expected. In contrast, volume of rainfall per month was similar between the two data collection periods.

**Table 1:** Summary of temperature, sunshine hours, and monthly rainfall volume retrieved from the Met Office for the 'spring/summer’ and 'autumn/winter’ data collection periods

	**Temperature: °C**				
**Data collection period**	**Mean**	**Min**	**Max**	**Sunshine hours**	**Monthly rainfall volume (mm)**
Spring/Summer	13.98	9.28	18.68	167.08	58.91
Autumn/Winter	6.28	3.03	9.50	64.35	56.11

### Main effects of season

Participants walked for on average 26.54 minutes (SD = 9.41) minutes immediately post-intervention, and for 19.91 minutes (SD = 5.48) on average at six-month follow up. There were no significant seasonal differences in number of minutes walked. Mean scores for Theory of Planned Behaviour variables immediately post-intervention and at 6 months, according to season, can be found in Table 2. There was no significant main effect of season on attitude, subjective norm or PBC.

**Table 2:** Mean (SD) values and repeated measures ANCOVAs of objective walking behaviour and TPB variables

**Measure**	**Spring/Summer**	**Autumn/Winter**	**F (df)**	**p-value**
	**Mean (SD)**	**Mean (SD)**		
***Objective walking behaviour***				
Immediately post-intervention (t2)	26.41 (9.94)	26.60 (9.58)		
Six-months post-intervention (t4)	19.45 (5.73)	19.58 (5.69)	.417 (1,227)	.519
***Direct TPB Measures***				
Attitude (t2)	6.23 (0.97)	6.00 (1.33)		
Attitude (t4)	5.54 (1.42)	5.42 (1.53)	1.74 (1, 208)	.192
Subjective Norm (t2)	4.62 (2.01)	4.55 (1.91)		
Subjective Norm (t4)	4.89 (1.30)	4.80 (1.57)	.003 (1,208)	.958
PBC (t2)	5.91 (1.29)	5.88 (1.33)		
PBC (t4)	5.60 (1.38)	5.81 (1.77)	.307 (1,192)	.580
Intention (t2)	6.03 (1.57)	6.13 (1.56)		
Intention (t4)	5.55 (1.38)	5.35 (1.56)	.888 (1,199)	.347
***Control beliefs***				
Time (t1)	5.06 (1.76)	4.97 (1.73)		
Time (t4)	4.56 (1.65)	4.72 (1.71)	.042 (1,196)	.837
Work and family commitments (t1)	3.68 (2.04)	3.59 (2.11)		
Work and family commitments (t4)	3.48 (1.87)	3.38 (1.85)	.041 (1, 196)	.839
Weather (t1)	4.53 (1.45)	3.06 (1.61)		
Weather (t4)	4.39 (1.38)	4.15 (1.32)	19.46 (1,191)	<.001
Pain (t1)	4.54 (2.35)	4.62 (2.31)		
Pain (t4)	4.58 (2.23)	4.94 (2.29)	3.77 (1,196)	.053
Threatening environments (t1)	6.28 (1.25)	6.10 (1.55)		
Threatening environments (t4)	6.03 (1.46)	6.10 (1.39)	2.156 (1,206)	.144
***Perceived power of control beliefs***				
Time (t1)	5.77 (1.61)	5.90 (1.42)		
Time (t4)	5.72 (1.46)	6.06 (1.21)	.000 (1,192)	.994
Work and family commitments (t1)	2.91 (1.56)	3.07 (1.68)		
Work and family commitments (t4)	2.90 (1.46)	2.54 (1.27)	3.115 (1,190)	.079
Weather (t1)	5.74 (1.66)	6.05 (1.29)		
Weather (t4)	5.50 (1.41)	5.88 (1.06)	1.021 (1,192)	.314
Pain (t1)	3.69 (1.86)	3.82 (1.83)		
Pain (t4)	3.67 (1.61)	3.18 (1.67)	2.397 (1,189)	.123
Threatening environments (t1)	1.85 (1.24)	2.32 (1.66)		
Threatening environments (t4)	2.15 (1.35)	1.90 (1.23)	.024 (1,198)	.877

Repeated measures ANCOVAs showed there was no effect of season on control beliefs relating to free time, work and family commitments, walking through threatening areas or pain. A significant main effect of season on control beliefs relating to good weather (i.e. there is likely to be good weather over the next 7 days) was found [F (1, 191) = 19.46, p = <.001]. However, the effect of season on the perceived power of this factor was non-significant (p = .314).

There was a significant main effect of intervention group on participants beliefs about whether they will walk through threatening areas (F (1, 206) = 4.06, p = .045) with the intervention group reporting a higher likelihood of walking through threatening areas. There was a significant main effect of intervention group on the perceived power of the control belief “I will have a lot of work and family commitments over the next 7 days” (F (1,190) = 4.847, p = .029).

### Sensitivity analysis

Mean pedometer step counts was 7492 (SD = 3173.8) immediately post-intervention, and 7677 (SD = 3116.7) at six months post-intervention. Repeated measures ANCOVA showed that there was no significant effect of season on pedometer step counts (p = .360).

### Moderator analyses

#### Predictors of objective walking behaviour

Season and TPB variables (intention and PBC) did not predict objective walking behaviour immediately post-intervention (t2) or at 6 months follow up (t4), according to hierarchical regression analysis. The inclusion of the interaction between season and PBC, or season and intention, did not contribute significantly to the prediction of objective walking behaviour at either t2 or t4 (p = .601 and p = .600, respectively).

### Predictors of intentions to walk more

The TPB variables at t2 explained 41.1% of the variance in intentions immediately post-intervention (p < .001). See Table 3. PBC was the only significant predictor in this model (β = .598, p < .001). Season did not predict intentions to walk more at time 2, and the inclusion of the interaction terms failed to significantly improve the model. At time 4 the TPB variables explained 47.4% of the variance in intentions (P < .001). See Table 4. Both attitude (β = .290) and PBC (β = .476) contributed unique variance to the model at this time point (both p < .001). The addition of season in to the model did not predict intentions to walk more over and above the contribution of intervention group and demographic variables. Interactions of season and TPB variables did not predict intentions to walk more.

**Table 3:** Hierarchical regression analysis of predictors of intentions to walk more immediately post-intervention

**Step**	**Variable entered**	**Beta**					**R**^**2**^	**Adjusted R**^**2**^	**Change in R**^**2**^	**P-value**
		**Step 1**	**Step 2**	**Step 3**	**Step 4**	**Step 5**				
		**Time 2 intention (n = 269)**								
1	Intervention group	.161	.167	.171	.079	.065	.026**	.022**	.026**	.008
2	Age		.027	.027	.049	.052	.039	.013	.013	.753
	Gender		-.017	-.019	-.011	-.011				
	BMI		-.026	-.027	-.021	-.021				
	Ethnic Group		.063	.061	.064	.054				
	Employment Status		.048	.048	-.006	-.012				
	Education Level		.050	.051	.044	.041				
3	Season			.015	-.014	-.022	.039	.009	.000	.819
4	T2 Attitude				.051	.042	.411***	.385***	.372***	<.001
	T2 Subjective Norm				-.011	-.052				
	T2 PBC				.598***	.570				
5	Season* Attitude					.043	.423	.391	.012	.155
	Season * SN					.089				
	Season * PBC					.057				

*** P < 0.001 (2-tailed), **p < 0.01, *p < 0.05.

**Table 4:** Hierarchical regression analysis of predictors of intentions to walk more at 6 months post-intervention

**Step**	**Variable entered**	**Beta**					**R**^**2**^	**Adjusted R**^**2**^	**Change in R**^**2**^	**P-value**
		**Step 1**	**Step 2**	**Step 3**	**Step 4**	**Step 5**				
		**Time 4 intention (n = 178)**								
1	Intervention group	.102	.093	.087	.010	.009	.010	.005	.010	.178
2	Age		.074	.073	-.003	.001	.030	-.010	.020	.753
	Gender		-.002	-.001	.074	.074				
	BMI		-.119	-.118	-.062	-.066				
	Ethnic Group		.002	.001	-.088	-.091				
	Employment Status		-.036	-.035	.045	.042				
	Education Level		-.001	<.001	.023	.027				
3	Season			.016	.068	.062	.030	-.016	<.001	.842
4	T4 Attitude				.290***	.262*	.474***	.439***	.444***	<.001
	T4 Subjective Norm				.056	.139				
	T4 PBC				.476***	.431**				
5	Season* Attitude					.035	.477	.432	.004	.772
	Season * SN					-.105				
	Season * PBC					.057				

***p < 0.001, **p < 0.01, *p < 0.05.

## Discussion

The present study did not find any significant differences in time spent walking, measured objectively using pedometers, between spring/summer and autumn/winter seasons. Moderator analyses also failed to show that season moderated the prediction of objective walking behaviour. Furthermore, in this study season did not significantly alter attitudes, subjective norm, PBC or intentions regarding walking behaviour. However, we did find a significant interaction between season and control beliefs regarding the weather, suggesting weather is a barrier.

### Strengths and weaknesses

This study is the first to our knowledge that investigated the impact seasonality has on objective walking behaviour as part of a trial. Previous research in the area has typically compared walking in the summer and winter months e.g. January and July with the most extreme weather patterns, to demonstrate the influence of seasonality. By analysing data collected as part of an RCT, over a 16 month period encompassing two autumn/winter and two spring/summer seasons, the present study was able to obtain a more naturalistic picture of the impact of seasons as a whole on behaviour, rather than the most extreme months. As the main aim of the RCT was to evaluate the effectiveness of an intervention to promote walking in primary care, deliberate sampling of data in summer and winter months was not conducted. Therefore there were unequal seasonal groups at each time point.

Particular strengths of the current research include its large sample size and use of a non-volunteer clinical population, both of which are in contrast to that of other studies in this area [13-16,19]. The present study obtained objective measures of walking from a pedometer that has been found to be an accurate and reliable measurement instrument with people in free-living environments [31] so was suitable for use with the patients involved in the current study. This study is also the first to investigate whether there are seasonal variations in theoretical determinants of objective walking behaviour.

### Comparison with the current literature

The findings of the present study are at odds with much previous research on this subject as we failed to find seasonal variability in objective walking behaviour. There are two likely explanations for this discrepancy in findings.

First, the current study employed a randomised controlled trial with recruitment over the entire period of the year in contrast to previous studies have employed longitudinal designs with deliberate sampling of pedometer data in summer and winter months. It is possible that weather and seasonal variations had less influence on participants recruited to the trial, as the weather was not so extreme. On a related point, the fact that the study was conducted in the UK rather than countries with more extreme weather such as Canada may have resulted in less seasonal variation than has typically be observed.

Second, participants were recruited to the present study on the basis that they wanted to increase their physical activity, and that they were inactive and had a chronic condition that would benefit from increases in physical activity. Therefore it could be assumed that they were committed to the study and were willing to continue to walk in spite of inclement weather associated with the autumn/winter months. Furthermore, the participants included in the above studies, with the exception of Chan et al. [19], were non-clinical populations who were often not inactive and may have less motivated to increase their physical activity.

Third, the pedometers used in the present study were programmed to record only moderate-vigorous activity. A recent study has found that light intensity activity was open to seasonal influence with lower levels of light intensity activity reported in winter compared to the other three seasons [16], there was no significant effect of season on moderate-vigorous activity in that sample. Given that previous studies in this area have assessed total physical activity as measured by pedometer [14,15,19], without specifying an intensity level, it is possible that the seasonal differences found in those studies could be attributed to changes in light intensity activity only.

The intervention reported by Chan et al. [19] was based on Social Cognitive Theory; however the authors did not attempt to evaluate the impact of seasonality on the theoretical variables underlying the intervention. In contrast the present study aimed to evaluate the effect of seasonality on the theoretical variables underlying the walking intervention. As such direct Theory of Planned Behaviour (TPB) variables were measured in the present study; attitude (an individual’s evaluation of the target behaviour), subjective norm (an individual’s subjective judgement concerning whether significant others would want them to perform the behaviour) and PBC (an individual’s perception of the level of control they have over performing the behaviour). These three variables are considered direct predictors of intentions to perform the behaviour, with intention and PBC considered direct predictors of behaviour itself. In the present study we did not find a significant impact of season on these directly measured TPB constructs i.e. attitude, intention, PBC and subjective norm.

The direct predictors of intentions and behaviour mentioned previously (i.e. attitude, subjective norm, PBC) are in turn influenced by indirect belief-based constructs. For instance, attitudes are determined by beliefs concerning the likely outcomes of the behaviour and an evaluation of these outcomes (behavioural beliefs) and subjective norms are determined by beliefs regarding the normative expectations of others and motivation to comply (normative beliefs). Beliefs regarding the presence of factors that may facilitate or inhibit the performance of the behaviour, in combination with the perceived power of these factors, give rise to control beliefs. As PBC has previously been shown to be the strongest predictor of intentions to walk more (26), and actual walking behaviour (27–28), the present study specifically examined the influence of season on the control beliefs underpinning PBC.

In the present study we found that seasonality had a significant impact on control beliefs related to weather. In other words participants expected the weather to be worse in the autumn/winter than the spring/summer. However, this control belief was not sufficient to bring about changes in the direct PBC construct measured in this study. A possible explanation for this is that season did not significantly affect the perceived power of this control belief (i.e. good weather will make it much easier to walk) in the present study. Given that control beliefs influence direct measures of PBC only when working in combination with their associated perceived power, it understandable that this did not impact on participants perceived behavioural control (PBC), and in turn their behaviour.

## Conclusions

The present study failed to demonstrate an effect of seasonality on objective walking behaviour in a randomised controlled trial of an intervention to promote walking in primary care, in contrast to the current literature on seasonality and walking behaviour. Although seasonality might influence trials in terms of recruitment of study participants [18] it does not appear to have the same influence on the outcomes evaluated by the trial. This study suggests therefore that the effects of seasonality are not large and have limited implications in terms of the conduct of randomised controlled trials. As such seasonality is unlikely to have biased the results of the previous intervention studies.

This is the first study to our knowledge that has investigated the influence of seasonality during a randomised controlled trial. As such it is recommended that this study is replicated, with a larger number of participants, to give further evidence regarding the relationship between season and objectively measured walking behaviour. We also recommend that future research investigates this issue in trials of walking interventions with different populations, in order to ascertain whether the findings of the present study are at odds with that of previous research due to differences in the population under investigation or study design.

## Ethical approval

Ethical approval for the present study was given by Coventry University Ethics Committee and Birmingham East, North and Solihull NHS Research Ethics Committee (Reference: 09/H1206/116).

## Abbreviations

PA: Physical activity; PBC: Perceived behavioural control; TPB: Theory of planned behaviour; ANCOVA: Analysis of covariance; RCT: Randomised controlled trial; BMI: Body mass index.

## Competing interests

The authors declare that they have no competing interests.

## Authors’ contributions

SLW coordinated the study, including producing study procedures, and led on statistical analysis and writing of this manuscript. DPF initially conceived and designed the study and contributed advice on statistics and to the writing of this manuscript. Both authors read and approved the final manuscript.

## Pre-publication history

The pre-publication history for this paper can be accessed here:

http://www.biomedcentral.com/1471-2458/14/120/prepub
